# Functional Mutation of Multiple Solvent-Exposed Loops in the *Ecballium elaterium* Trypsin Inhibitor-II Cystine Knot Miniprotein

**DOI:** 10.1371/journal.pone.0016112

**Published:** 2011-02-18

**Authors:** Richard H. Kimura, Douglas S. Jones, Lei Jiang, Zheng Miao, Zhen Cheng, Jennifer R. Cochran

**Affiliations:** 1 Department of Radiology, Molecular Imaging Program at Stanford (MIPS), Cancer Center, Bio-X Program, Stanford University, Stanford, California, United States of America; 2 Department of Bioengineering, Cancer Center, Bio-X Program, Stanford University, Stanford, California, United States of America; University of Crete, Greece

## Abstract

**Background:**

The *Ecballium elaterium* trypsin inhibitor (EETI-II), a 28-amino acid member of the knottin family of peptides, contains three interwoven disulfide bonds that form multiple solvent-exposed loops. Previously, the trypsin binding loop of EETI-II has been engineered to confer binding to several alternative molecular targets. Here, EETI-II was further explored as a molecular scaffold for polypeptide engineering by evaluating the ability to mutate two of its structurally adjacent loops.

**Methodology/Principal Findings:**

Yeast surface display was used to engineer an EETI-II mutant containing two separate integrin binding epitopes. The resulting knottin peptide was comprised of 38 amino acids, and contained 11- and 10-residue loops compared to wild-type EETI-II, which naturally contains 6- and 5-residue loops, respectively. This knottin peptide bound to α_v_β_3_ and α_v_β_5_ integrins with affinities in the low nanomolar range, but bound weakly to the related integrins α_5_β_1_ and α_iib_β_3_. In addition, the engineered knottin peptide inhibited tumor cell adhesion to vitronectin, an extracellular matrix protein that binds to α_v_β_3_ and α_v_β_5_ integrins. A ^64^Cu radiolabeled version of this knottin peptide demonstrated moderate serum stability and excellent tumor-to-muscle and tumor-to-blood ratios by positron emission tomography imaging in human tumor xenograft models. Tumor uptake was ∼3–5% injected dose per gram (%ID/g) at one hour post injection, with rapid clearance of probe through the kidneys.

**Conclusions/Significance:**

We demonstrated that multiple loops of EETI-II can be mutated to bind with high affinity to tumor-associated integrin receptors. The resulting knottin peptide contained 21 (>50%) non-native amino acids within two mutated loops, indicating that extended loop lengths and sequence diversity were well tolerated within the EETI-II scaffold. A radiolabeled version of this knottin peptide showed promise for non-invasive imaging of integrin expression in living subjects. However, reduced serum and metabolic stability were observed compared to an engineered integrin-binding EETI-II knottin peptide containing only one mutated loop.

## Introduction

Cystine-knot miniproteins, also known as knottins, are small polypeptides (20–60 amino acids) that have an interwoven disulfide-bonded framework, triple-stranded β-sheet fold, and possess one or more solvent exposed loops that mediate binding to diverse targets [1], [2]. Knottin family members, which include toxins, antimicrobials, ion channel inhibitors, and protease inhibitors, share little sequence homology apart from their core cysteine residues [3]–[5]. As a result, binding epitopes have been introduced into knottin peptides to impart them with new molecular recognition properties without abolishing their three-dimensional fold [6]–[12]. The *Ecballium elaterium* trypsin inhibitor (EETI-II) knottin contains three disulfide bonds and binds to and inhibits trypsin through a single 6-amino acid loop [13], [14]. In pivotal studies, EETI-II was used as a molecular scaffold by rationally substituting this trypsin binding loop (PRILMR) with grafted biologically-active peptides against targets such as elastase, thrombopoietin, and integrins [7], [9], [10].

Integrins are a family of α/β heterodimeric adhesion receptors that have distinct ligand binding specificities and cell signaling properties [15]. Non-invasive molecular imaging agents that target tumor-related integrin receptors will play an important role in earlier cancer detection, disease staging, and management [16], [17]. We recently used yeast surface display, a combinatorial method, to identify EETI-II-based knottin peptides that bound with high (low nM) affinity to α_v_β_3_/α_v_β_5_ or α_v_β_3_/α_v_β_5_/α_5_β_1_ integrins [8], which are overexpressed on tumors or their neovasculature and mediate angiogenesis and metastasis [18]–[21]. In mouse tumor models, radiolabeled versions of these integrin-binding knottin peptides exhibited high tumor uptake with low background in non-target tissue (i.e. liver and kidney) [22], [23].

While our previous study focused on combinatorial libraries of the EETI-II trypsin binding loop (Loop 1), additional work by our group demonstrated high tolerance of length and sequence diversity in other EETI-II loops [24]. In particular, a loop containing the sequence GPNGF (Loop 3) accommodated broad sequence diversity and tolerated a wide range of loop lengths beyond its original 5 amino acids. In the current study, our goal was to further explore the utility of knottins as molecular scaffolds for polypeptide engineering by evaluating the ability to mutate two structurally-adjacent loops within EETI-II. We used yeast surface display to engineer an EETI-II-based knottin peptide that contains an 11-amino acid sequence in place of Loop 1, and a 10-amino sequence in place of Loop 3. As a model system, each engineered loop contained a separate Arg-Gly-Asp (RGD) integrin-binding motif [25], whose flanking residues were optimized to bind with high affinity to tumor-associated integrin receptors. We measured the binding affinity and specificity of this engineered knottin peptide against cell lines expressing particular integrin receptors. In addition, we radiolabled this knottin peptide and evaluated its ability to target tumors in mouse xenograft models. Importantly, we demonstrated that multiple loops of a knottin peptide, comprising 21 non-native amino acid residues, can be engineered to bind with high affinity to integrin receptors, and the resultant peptide can be used as a probe for non-invasive molecular imaging applications. These findings expand the potential of the cystine-knot scaffold for future studies where multiple knottin loops can be simultaneously engineered to bind to exogenous targets. Such capability will be important, for example, in engineering binders against molecular targets which require an increased surface area to achieve high affinity interactions.

## Results

### Engineering Knottin Peptides Containing Two Separate Integrin Binding Loops

**Figure 1:**
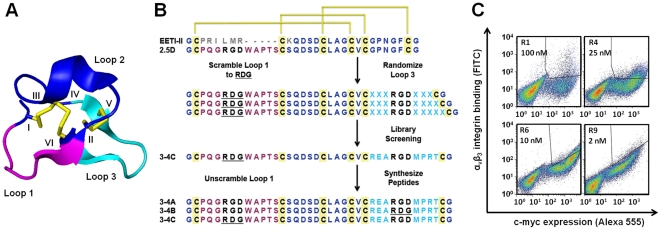
Summary of knottin engineering. (**A**) Three dimensional structure of wild-type EETI-II (pbd: 2it7, ref [13]) showing Loop 1, previously engineered for high affinity α_v_β_3_ integrin binding (magenta), Loop 2 (dark blue), and Loop 3 (cyan). Cysteines I to VI are labeled and shown in yellow. (**B**) Strategy for knottin engineering. The primary sequences of wild-type EETI-II and mutant 2.5D are shown with disulfide connectivities indicated in yellow. The RGD motif in Loop 1 was scrambled to RDG and three different Loop 3 libraries were created and screened for high affinity to α_v_β_3_ integrin. After nine rounds of library screening by FACS the predominant mutant, knottin 3-4C, is shown. Three different variants 3-4A: (RGD/RGD), 3-4B: (RGD/RDG), and 3-4C: (RDG/RGD) were synthesized and folded. (**C**) FACS density dot plots showing the library sort progression for enrichment of improved α_v_β_3_ integrin binders. R denotes the sort round, and the concentration of α_v_β_3_ integrin is indicated. Actual sort gates are shown as polygons in the upper right quadrant.

Previously, we used yeast surface display to engineer a high affinity integrin binding sequence into Loop 1 of EETI-II (Figure 1A, magenta) [8]. This mutant was termed EETI-II 2.5D. In the current study, our goal was to explore the potential to introduce an additional, but distinct, integrin-binding epitope within a structurally-adjacent knottin loop. EETI-II Loop 3 (Figure 1A, cyan) was chosen as our previous work suggested that this loop was more amenable to mutation compared to Loop 2 [24]. First, we scrambled the RGD sequence in EETI-II 2.5D (PQGRDGWAPTS), abolishing its function, so that we could evolve a separate integrin binding epitope within Loop 3. Second, combinatorial libraries were created in which EETI-II Loop 3 (sequence GPNGF) was substituted with XXXRGDXXX, XXXRGDXXXX, and XXXRGDXXXXX, where X can be any amino acid (Figure 1B and Table S1). These loop libraries of 9, 10, and 11 amino acids were chosen based on our experience with engineering optimized integrin-binding knottin peptides [8], [11], [12]. EETI-II mutants were displayed on the surface of yeast as fusions to the agglutinin mating proteins under the control of a galactose promoter, and contained a C-terminal cMyc epitope tag for detection and quantification of knottin expression levels using an anti-cMyc antibody. The yeast-displayed EETI-II libraries were pooled, and were screened using high-throughput fluorescent-activated cell sorting (FACS) to isolate mutants that were well-expressed on the yeast cell surface and bound with high affinity to detergent-solubilized α_v_β_3_ integrin. The initial library contained a small fraction of clones that bound to 100 nM α_v_β_3_ integrin (Figure 1C), demonstrating that specific RGD flanking sequences are critical for high affinity integrin binding. Integrin-binding yeast were isolated, cultured, and induced for knottin expression, and the sorting process with α_v_β_3_ integrin was repeated. Nine rounds of FACS were used to isolate mutants that bound with high affinity to α_v_β_3_ integrin (Table S2). In later sort rounds the integrin concentration was lowered to 2 nM, and a diagonal sort gate was used to isolate clones that bound the highest levels of α_v_β_3_ integrin for a given level of yeast expression (Figure 1C). A predominant clone, 3-4C (Loop 1: PQGRDGWAPTS, Loop 3: REARGDMPRT), was isolated in the final round of sorting after conducting an “off-rate” screen by incubating the library with 2 nM α_v_β_3_ integrin, followed by a 4 hour unbinding step in the presence of soluble EETI-II 2.5D competitor. We synthesized three permutations of this knottin peptide consisting of one or two functional integrin binding loops: 1) 3-4A (Loop 1: RGD/Loop 3: RGD), 2) 3-4B (Loop 1: RGD/Loop 3: RDG), and 3) 3-4C (Loop 1: RDG/Loop 3: RGD) (Figure 1B). These knottin peptides were folded and purified as previously described [8], and their masses were confirmed by matrix-assisted laser desorption/ionization time-of-flight (MALDI-TOF) mass spectrometry (Figure S1 and Table S3).

### Binding of Engineered Knottin Peptides to U87MG Glioblastoma Cells

**Figure 2:**
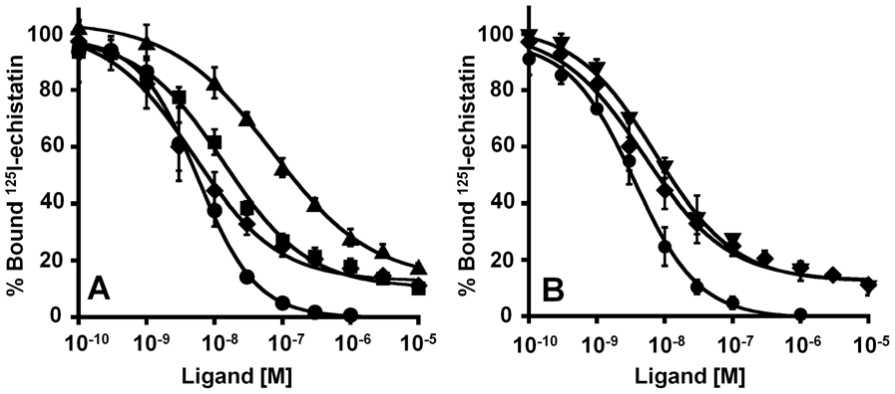
Competition binding to integrin receptors expressed on U87MG cells. Varying concentrations of unlabeled knottin peptides or DOTA-conjugated knottin 3-4A were incubated with ^125^I-echistatin and allowed to compete for binding to integrin receptors expressed on the surface of U87MG cells. Percent of ^125^I-echistatin bound to the cell surface is plotted versus the concentration of unlabeled knottin (**A**) 3-4A (⧫), 3-4B (▴), 3-4C (▪), or (**B**) DOTA-knottin 3-4A (▾) and unlabeled knottin 3-4A (⧫). Echistatin (•) was used as a positive control to compare binding data from different experiments. The binding curves for the knottin peptides did not reach full inhibition because ^125^I-echistatin binds with broad specificity to multiple integrins expressed on U87MG cells. Data shown are the average of triplicate values and error bars represent standard deviations. IC_50_ values are summarized in Table 1.

The engineered knottin peptides were tested for their ability to compete with ^125^I-echistatin for binding to integrin receptors expressed on U87MG glioblastoma cells, which express ∼10^5^ α_v_β_3_ integrin receptors per cell [26]. Echistatin, a polypeptide found in snake venom, binds to multiple integrin receptors with high affinity [8], [27] and was used as a positive control. The knottin peptide 3-4C (RDG/RGD) bound to U87MG cells with an IC_50_ value of 15±3 nM (Figure 2 and Table 1). In comparison, the knottin peptides 3-4A (RGD/RGD) and 3-4B (RGD/RDG) bound to U87MG cells with IC_50_ values of 5±2 nM and 68±8 nM, respectively, relative to echistatin, which bound with an IC_50_ value of 4.9±0.1 nM (Figure 2 and Table 1). EETI-II 2.5D (Figure 1B), which was used as a starting point for the current knottin engineering study, was previously found to have an IC_50_ of 19±6 nM [8]. Mutant 3-4B (RGD/RDG) exhibited a ∼3.5-fold decrease in binding compared to EETI-II 2.5D, most likely due to conformational perturbations that result when Loop 3 is mutated from 5 to 10 amino acids. However, these substantial changes in the length and composition of Loop 3 do not abolish the ability of Loop 1, which contains the sequence PQGRDGWAPTS from EETI-II 2.5D, to bind to integrins with high affinity. In addition, the weaker binding affinity observed with mutant 3-4B (RGD/RDG) compared to 3-4A (RGD/RGD) highlights the interdependence of the loops in integrin binding, while the similarities in binding affinity of mutant 3-4A (RGD/RGD) and 3-4C (RDG/RGD) suggests that the newly engineered Loop 3 contributes to the majority of the binding.

**Table 1:** Summary of IC_50_ values from competition binding and cell adhesion assays.

Ligand	Binding Assay (nM)					Cell Adhesion (nM)	
	U87MG	K562 (wt)	K562(α_v_β_3_)	K562(α_v_β_5_)	K562(α_iib_β_3_)	Vitronectin	Fibronectin
Echistatin	4.9±0.1	0.7±0.2	5±1	2.0±0.2	5±3	1.4±0.1	21±5
3-4A (RGD/RGD)	5±2	500±200	7±4	7±1	300±100	9±3	>1 µM
3-4B (RGD/RDG)	68±8	n/d	n/d	n/d	n/d	n/d	n/d
3-4C (RDG/RGD)	15±3	700±200	5±1	13±5	400±100	15±2	>1 µM
DOTA-3-4A	8±1	n/d	n/d	n/d	n/d	n/d	n/d

Data are reported in nM unless otherwise stated. n/d indicates not determined.

### Integrin-Binding Specificity of Engineered Knottin Peptides

U87MG cells have been shown to express several different integrin receptor subtypes, including α_v_β_3_, α_v_β_5_, and α_5_β_1_ [28]. Therefore, to probe the integrin binding specificity of the engineered knottin peptides, competition binding assays were performed with ^125^I-echistatin and K562 leukemia cells, which naturally express α_5_β_1_ integrin, or K562 cells that have been stably transfected to express α_v_β_3_, α_v_β_5_, or α_iib_β_3_ integrin receptors [29]. Echistatin, which has been previously shown to bind to α_v_β_3_, α_v_β_5_, α_5_β_1_, and α_iib_β_3_ integrins [8], [27], was used as a positive control, and bound strongly to all cell types tested (Figure S2). Engineered knottin peptides 3-4A (RGD/RGD) and 3-4C (RDG/RGD) bound with weak affinity to untransfected K562 cells expressing α_5_β_1_ integrin, with IC_50_ values of 500±200 nM and 700±200 nM, respectively (Figure S2A and Table 1). In contrast, knottin peptides showed increased binding affinity to K562 cells expressing α_v_β_3_ or α_v_β_5_ integrins, with IC_50_ values approaching that of echistatin (Figure S2B-C and Table 1). Finally, we determined that the engineered knottin peptides 3-4A and 3-4C bound weakly to K562 cells expressing α_iib_β_3_ integrin, with IC_50_ values of 300±100 nM and 400±100 nM, respectively (Figure S2D and Table 1). These IC_50_ values are similar to those obtained for untransfected K562 cells expressing α_5_β_1_ integrin, but are two orders of magnitude weaker than binding to K562 cells expressing α_v_β_3_ or α_v_β_5_integrins.

### Engineered Knottin Peptides Inhibit Tumor Cell Adhesion to Vitronectin

**Figure 3:**
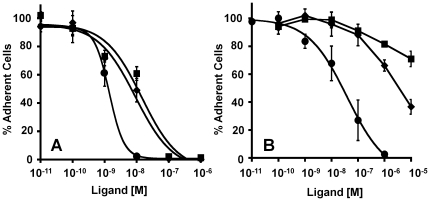
Inhibition of integrin-dependent tumor cell adhesion. (**A**) Vitronectin or (**B**) fibronectin coated strips were incubated with U87MG cells for 2 h with varying concentrations of echistatin (•), knottin 3-4A (⧫), and knottin 3-4C (▪). Adherent cells remaining after several wash steps were quantified with crystal violet staining by absorbance at 600 nm. Values were normalized against uncoated wells and wells containing no competing peptide. Data shown are the average of three replicates performed on different days and error bars represent standard deviations. IC_50_ values are summarized in Table 1.

The ability to block tumor cells from adhering to extracellular matrix proteins is important for treatment strategies designed to inhibit angiogenesis and metastasis. Thus, we measured the ability of engineered knottin peptides to block U87MG cell adhesion to vitronectin- and fibronectin-coated microtiter plates. Vitronectin is a natural ligand for several integrins, including α_v_β_3_ and α_v_β_5_, while the α_5_β_1_ integrin receptor is selective for fibronectin [15], [30]. The engineered knottin peptides strongly inhibited U87MG cell adhesion to vitronectin in a dose-dependent manner with IC_50_ values in the low nM range (Figure 3A and Table 1), reflecting their binding affinity to α_v_β_3_ and α_v_β_5_ integrins. In contrast, the engineered knottin peptides only weakly inhibited U87MG cell adhesion to fibronectin, with IC_50_ values greater than 1 µM (Figure 3B and Table 1), reflecting their much weaker binding to α_5_β_1_ integrin. Echistatin, which binds to α_5_β_1_, α_v_β_3_, α_v_β_5_, and α_iib_β_3_ integrins, was used as a positive control and inhibited U87MG cell adhesion to both vitronectin- and fibronectin-coated plates in a dose-dependent manner with IC_50_ values in the low nM range (Figure 3).

### Synthesis and Characterization of DOTA-knottin 3-4A and ^64^Cu-DOTA-knottin 3-4A

**Figure 4:**
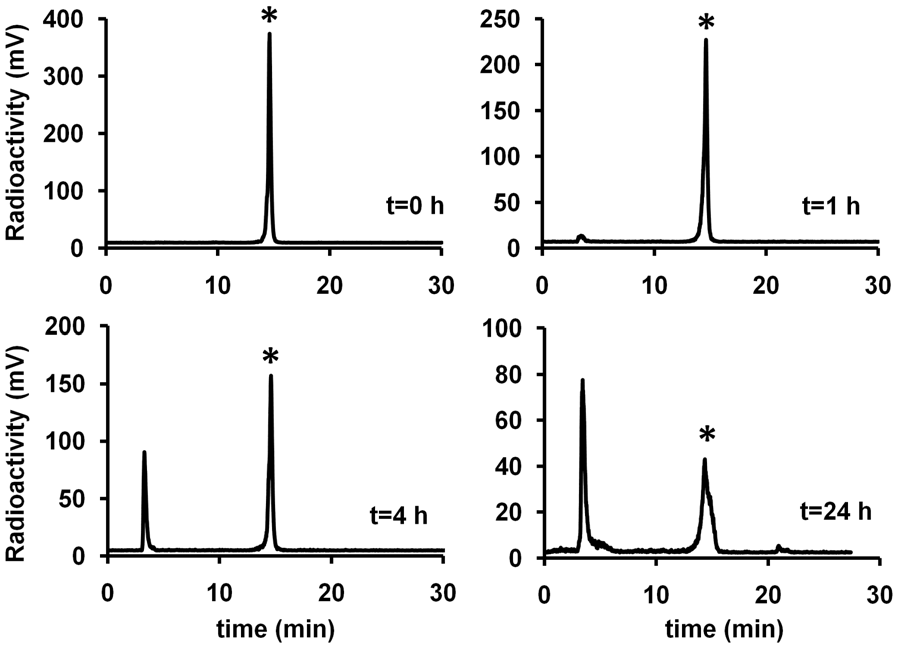
Serum stability of ^64^Cu-DOTA-knottin 3-4A. ^64^Cu-DOTA-knottin 3-4A was incubated with mouse serum for up to 24 h at 37°C. Representative HPLC traces are shown with radioactive signal (mV) plotted as a function of time. Intact ^64^Cu-DOTA-knottin 3-4A (*) elutes at approximately 15 minutes. The amount of probe remaining at 1, 4, and 24 h was quantified from the area under the entire peak to be 96%, 55%, and 30%, respectively.

The knottin peptide 3-4A was site-specifically conjugated to 1,4,7,10-tetra-azacyclododecane-N,N',N'',N'''-tetraacetic acid (DOTA) through its N-terminal amino group using an N-hydroxysuccimide ester DOTA derivative. The resulting DOTA-conjugated knottin peptide was purified by reversed-phase HPLC and characterized by MALDI-TOF-MS (Figure S1 and Table S3). Competition binding assays were performed with U87MG cells and ^125^I-echistatin as described above for the unconjugated peptides (Figure 2B). The binding affinity of DOTA-knottin 3-4A (IC_50_ = 8±1 nM) is essentially unchanged compared to the unmodified peptide (IC_50_ = 5±2 nM), showing that addition of DOTA to the N-terminus did not interfere with integrin binding. The DOTA-knottin 3-4A peptide was radiolabeled with ^64^Cu, and tumor cell binding and uptake were measured using U87MG glioblastoma cells (Figure S3). The target specificity of ^64^Cu-DOTA-knottin 3-4A was measured by blocking studies using an unlabeled integrin binding peptide (c(RGDyK)) that binds to the same epitope on the integrin receptor. Incubation of ^64^Cu-DOTA-knottin 3-4A with an excess of unlabeled c(RGDyK), resulted in a significant decrease in cell binding (Figure S3). Next, the serum stability of ^64^Cu-DOTA-knottin 3-4A was measured after incubation in 50% mouse serum at 37°C for up to 24 hours (Figure 4). After incubation for 1 hour in mouse serum, the radiolabeled peptide was ∼95% intact and slowly degraded over time to ∼30% after incubation in serum for 24 hours.

### MicroPET Imaging of ^64^Cu-DOTA-knottin 3-4A in U87MG Xenografts

**Figure 5:**
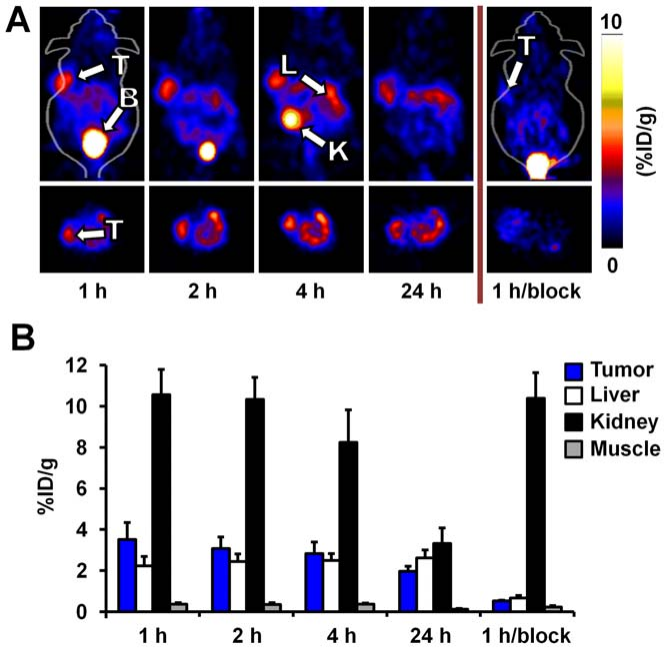
MicroPET imaging of U87MG tumor xenografts. (**A**) Representative microPET scans (coronal images, top; transverse images, bottom) of U87MG xenografts (n = 3) after injection of ^64^Cu-DOTA-knottin 3-4A alone or co-injected with a large molar excess of unlabeled c(RGDyK) pentapeptide (1 h/block). The letters B, K, L, and T represent bladder, kidney, liver and tumor, respectively. (**B**) The mean %ID/g for tumor, liver, kidney, and muscle uptake were quantified on images generated 1, 2, 4, and 24 h post injection. Error bars represent standard deviations of experiments performed in three mice.

Using non-invasive microPET imaging, we evaluated tumor and non-target tissue uptake and clearance of ^64^Cu-DOTA-knottin 3-4A in mice bearing human U87MG xenografts. ^64^Cu-DOTA-knottin 3-4A rapidly accumulated at the tumor to levels of 3.51±0.83%ID/g after 1 h post injection and cleared at a rate of approximately 0.07%ID/g/h, resulting in a signal of 1.97±0.24%ID/g at 24 h post injection (Figure 5). Kidney uptake ranged from 10-12%ID/g at 1 h post injection and decreased to ∼3–4%ID/g after 24 h post injection. Liver uptake remained low around ∼2%ID/g throughout the imaging study. Target specificity of ^64^Cu-DOTA-knottin 3-4A in mouse tumor models was confirmed by co-injecting a 1000-fold molar excess of unlabeled c(RGDyK), resulting in significantly reduced tumor uptake at 1 h post injection (0.53± 0.03%ID/g; p<0.05) (Figure 5).

### Biodistribution of ^64^Cu-DOTA-knottin 3-4A in U87MG Xenografts

**Figure 6:**
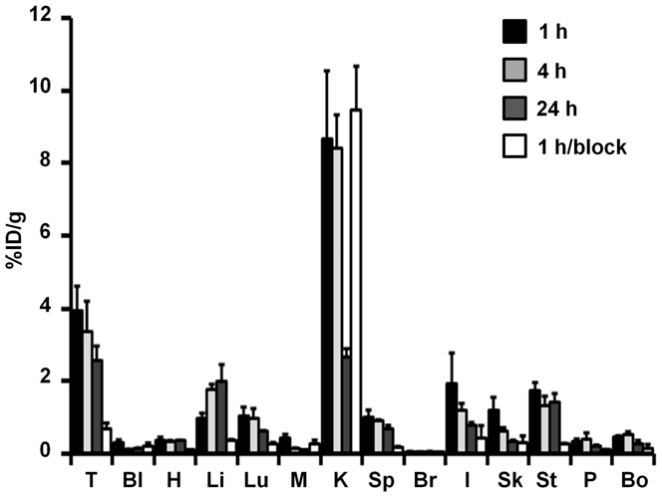
Biodistribution in U87MG tumor xenografts. Biodistribution of ^64^Cu-DOTA- knottin 3-4A in U87MG xenografts (n = 3) was measured in the tumor (T), blood (Bl), heart (H), liver (Li), lungs (Lu), muscle (M), kidneys (K), spleen (Sp), brain (Br), intestine (I), skin (Sk), stomach (St), pancreas (P), and bone (Bo). Data are presented as the %ID/g tissue ± SD (n = 3) after intravenous injection of ∼50–100 µCi probe at 1 h (black bars), 4 h (light grey bars), and 24 h (dark grey bars). To measure probe specificity, mice were injected with ^64^Cu-DOTA- knottin 3-4A and an excess of unlabeled competitor (c(RGDyK)), and tissue biodistribution was measured after 1 h (1 h/block, white bars). Error bars represent standard deviations of experiments performed in three mice.

The biodistribution of ^64^Cu-DOTA-knottin 3-4A was determined at 1, 4, and 24 h post injection in U87MG xenograft mice (Figure 6). Rapid tumor uptake was observed at 1 h post injection (3.92±0.70%ID/g). By measuring radioactivity in the tumor at 24 h post injection (2.56±0.40%ID/g), a radiotracer washout rate of 0.06%ID/g/h was calculated. These data are in overall agreement with microPET imaging data presented in Figure 5. In addition, tumor uptake was significantly inhibited in blocking studies where a 1000-fold molar excess of unlabeled c(RGDyK) was co-injected with the radiotracer (0.66±0.17%ID/g; p<0.05). These data further confirm the specificity of the ^64^Cu-DOTA-knottin 3-4A for integrin receptors expressed at the tumor site. ^64^Cu-DOTA-knottin 3-4A is cleared through the kidneys and a low residual radioactivity remained at 24 h post injection (2.67±0.22%ID/g), demonstrating that the kidneys do not retain high amounts of the radiotracer or its metabolic byproducts. Biodistribution analysis showed low accumulation of radioactivity in other non-target organs (Figure 6). Slight accumulation of radioactivity in the liver was observed from ∼1 to 2%ID/g at 1 to 24 h post injection, respectively. Tissues originating from the thoracic region exhibited low levels of background signal, while radioactivity measured in the lungs decreased from ∼1 to 0.6%ID/g from 1 to 24 h post injection. Heart and blood radioactivity measured ∼0.3%ID/g throughout the study. Radioactivity in the muscle tissue was also low (<0.4%ID/g). These data indicate that ^64^Cu-DOTA-knottin 3-4A has promising potential for molecular imaging of integrin expression in living subjects.

### In Vivo Stability of ^64^Cu-DOTA-knottin 3-4A

**Figure 7:**
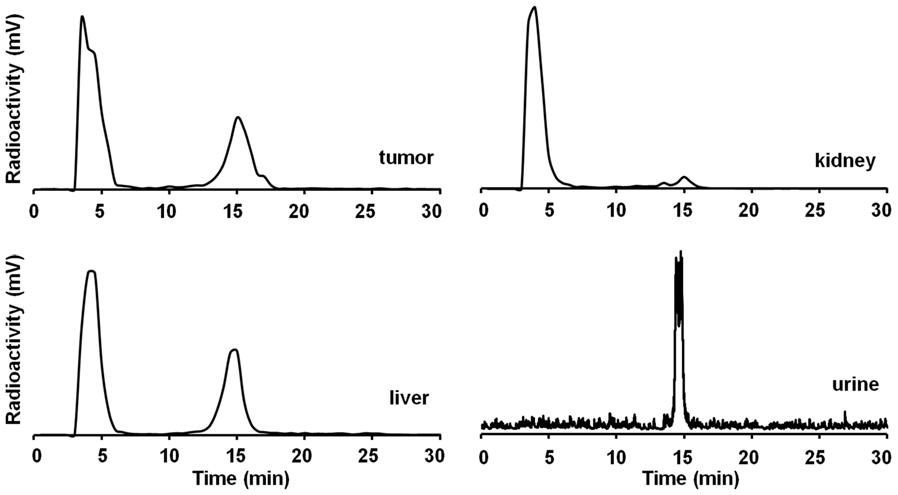
In vivo stability of ^64^Cu-DOTA-knottin 3-4A. Urine samples or homogenized tumor, kidney, or liver tissue were analyzed by radio-HPLC and gamma counting 1 h post injection. Representative HPLC traces are shown. The intact radiotracer elutes at approximately 15 minutes, while the major hydrophilic metabolites and free copper elute with the column flow through. Only limited quantities of urine were available from the mice, resulting in higher background noise for this sample.

Finally, metabolic stability of ^64^Cu-DOTA-knottin 3-4A in U87MG xenograft models was evaluated at 1 h post injection (Figure 7). Analysis of urine samples indicated that intact knottin radiotracer is excreted through the bladder, compared to the kidneys where a hydrophilic metabolite (or free copper) is responsible for the majority of the radioactive signal. Breakdown of the probe also occurs in the liver and tumor, with less than ∼40% of the intact radiotracer recoverable after 1 h.

## Discussion

The EETI-II miniprotein consists of at least three solvent accessible loops that are potential regions for amino acid substitution or randomization. Previous studies have introduced non-native epitopes into Loop 1 [6], [7], [9], [10] or Loop 3 [24], [31] of EETI-II to study structure/function effects or to create peptides with new molecular recognition properties. Recent work has shown that knottin peptides have promise as tumor-targeting probes for diagnostic applications across several molecular imaging modalities [22], [23], [32]–[38]. In the current study, we used yeast surface display to engineer EETI-II mutants with integrin-binding motifs substituted into both Loops 1 and 3, with several goals in mind. The first was to use this model system to explore the boundaries of significantly mutating a knottin scaffold for polypeptide engineering applications. A second goal was to compare this new peptide to an existing integrin-binding EETI-II mutant [22], [23], in terms of binding affinity, specificity, stability, tumor uptake and clearance, and tissue biodistribution for non-invasive molecular imaging applications.

Extracellular matrix proteins including vitronectin, fibronectin, osteopontin, laminin, and others, bind to particular integrin receptors through an RGD peptide motif [25]. The RGD sequence is constrained within a loop structure that presents a particular conformation and stereochemical arrangement of residues critical for optimal integrin binding affinity and specificity [39], [40]. Hence, simple substitution of EETI-II Loop 3 with several different loop sequences derived from the integrin-binding domain of fibronectin resulted in knottin peptides with weak binding to α_v_β_3_ integrin (data not shown). Similarly, our initial knottin RGD-loop libraries contained very few integrin binders, which were enriched over multiple rounds of screening. Although library screens were performed against α_v_β_3_ integrin, the knottin peptides exhibited high affinity binding to both α_v_β_3_ and α_v_β_5_ integrins, which are often co-expressed on tumors or the tumor vasculature [18], [19], and substantially weaker binding to the structurally-related α_5_β_1_ and/or α_iib_β_3_ integrins. Previously, to isolate knottin peptides with specificity only to α_iib_β_3_ integrin for inhibition of platelet aggregation, we interspersed positive sorts against α_iib_β_3_ integrin with negative sorts against α_v_β_3_ integrin, otherwise the isolated knottin peptides bound with high affinity to both integrins [11].

Substitution of EETI-II Loop 1 (normally 6 amino acids) with an 11-amino acid loop, and Loop 3 (normally 5 amino acids) with a 10-amino acid loop resulted in functional, high affinity integrin binding mutants. Both engineered loops were capable of binding to integrins with high affinity, as confirmed by measurements performed with knottins containing scrambled RDG control loops. Mutant 3-4B (RGD/RDG) exhibited decreased binding compared to EETI-II 2.5D on U87MG cells (68±8 nM versus 19±6 nM), indicating a ∼3-fold drop in affinity through introduction of a 10-amino acid loop in place of the GPNGF sequence of Loop 3. Mutant 3-4A (RGD/RGD) exhibited a ∼3-fold improvement in binding compared to mutant 3-4C (RDG/RGD) to U87MG cells (RGD/RGD: 5±2 nM versus RDG/RGD: 15±3 nM), and at least a 2-fold improvement in binding to K562-α_v_β_5_ cells (RGD/RGD: 7±1 nM versus RDG/RGD: 13±5 nM); however, binding to K562-α_v_β_3_ cells was similar (RGD/RGD: 7±4 nM versus RDG/RGD: 5±1 nM). Since U87MG cells express multiple integrin receptors, including α_v_β_3_ and α_v_β_5_, the enhanced binding of mutant 3-4A to these cells compared to mutant 3-4C most likely results from the contributions of α_v_β_5_ integrin. Despite the higher affinity of mutant 3-4A to U87MG cells compared to EETI-II 2.5D, we did not observe an improvement in its ability to inhibit cell adhesion to vitronectin (IC_50_: 9±3 nM versus 10±2 nM [8]).

In previous work, we established EETI-II 2.5D as a new agent for non-invasive imaging of integrin expression in living subjects. We showed that ^18^F- and ^64^Cu-labeled EETI-II 2.5D exhibited high tumor uptake, and low uptake in non-target tissue, including liver and kidneys [22], [23]. ^64^Cu-DOTA-knottin 3-4A exhibited comparable tumor uptake levels to ^64^Cu-DOTA-knottin 2.5D in U87MG xenograft models (3.51±0.83%ID/g versus 4.47±1.21%ID/g at 1 h post injection from microPET data), in accordance with their similar binding affinities. Radioactivity clearance from the tumor site from 1 to 24 h post injection was 0.07±0.02%ID/g/h for ^64^Cu-DOTA-knottin 3-4A, compared to 0.13±0.04%ID/g/h for ^64^Cu-DOTA-knottin 2.5D. High tumor uptake and rapid blood clearance led to favorable tumor-to-blood (T/B) and tumor-to-muscle (T/M) ratios for both probes. At 4 h post injection, the T/B and T/M ratios were 27±3 and 31±7, respectively, for ^64^Cu-DOTA-knottin 3-4A, and 25±3 (T/B) and 30±1 (T/M) for ^64^Cu-DOTA-knottin 2.5D. Liver uptake was low, with %ID/g values of ∼1–2%ID/g. The primary clearance route for ^64^Cu-DOTA-knottin 3-4A is the kidneys, with probe uptake values ranging from ∼8–12%ID/g at 1−4 h post-injection, and decreasing to ∼2–4%ID/g at 24 h. In comparison, ^64^Cu-DOTA-knottin 2.5D exhibited lower kidney uptake values of ∼3%ID/g at 4 post-injection, which were further reduced to ∼1%ID/g at 24 h post-injection [23]. Despite these differences, the tissue biodistibution of ^64^Cu-DOTA-knottin 3-4A is significantly improved compared to other polypeptide-based probes, which often show extremely high uptake and retention of ^64^Cu in the kidneys and liver [34]. For example, in a recent study ^64^Cu-labeled tumor-targeting peptides based on a scaffold from staphylococcal protein A (affibodies) had kidney and liver uptake values ranging from 40–240%ID/g and 5–12%ID/g, respectively, during measurements taken over 20 h post injection [41].

In previous studies, we used a ^64^Cu-labeled EETI-II-based knottin peptide containing a RDG sequence to measure non-specific probe uptake in U87MG tumor xenograft models (1.09±0.48%ID/g and 0.76±0.33%ID/g at 1 and 4 h post injection, respectively) [23]. Target specificity of ^64^Cu-DOTA-knottin 3-4A was measured by blocking experiments performed by co-injecting an excess of unlabeled c(RGDyK) competitor (Figure 5 and Figure 6). In addition, decreases in tumor uptake with ^64^Cu-DOTA-knottin 3-4A were seen at 1 h post injection (0.82±0.05%ID/g) in a MDA-MB-435 melanoma xenograft model (data not shown), which expresses lower levels of α_v_β_3_ integrin compared to U87MG xenograft tumors.

Myriad integrin-binding peptidomimetics containing a constrained RGD sequence have been developed as molecular imaging agents [16], [17], [42], [43]. Multivalent versions of these peptidomimetics have been created with the goal of improving integrin binding affinity and, hence, tumor uptake [44], [45]. In one example, c(RGDyK) dimers and higher order oligomers have been created by chemical conjugation through addition of a glutamate branching residue [46]. Competition binding of ^125^I-echistatin to U87MG cells with this dimeric ligand compared to monomeric c(RGDyK) showed a small increase in binding affinity when two RGD motifs were present (IC_50_ = 103±14 nM versus 203±32 nM). This 2-fold difference in binding affinity to U87MG cells is similar to the approximately 3-fold improvement we observed here with engineered knottin peptides containing two RGD motifs compared to one RGD motif (Table 1). In this previous study and in our current work, molecules with two RGD motifs exhibited a free energy of binding that was less than additive compared to the sum of individual RGD monomer contributions, indicating that the RGD motifs cannot both effectively engage integrin receptors due to steric constraints, or that strain or distortion in the ligand or cell membrane (i.e. to promote receptor conformation changes or clustering) causes substantial entropy loss upon binding [47],[48]. However, while we did not observe enhanced tumor uptake of radiolabeled knottins containing two RGD motifs compared to one, the ^64^Cu-labeled c(RGDyK) dimer was reported to exhibit a 2-fold-increase in tumor uptake compared to monomeric ^64^Cu-labeled c(RGDyK) [49], [50].

In more recent examples, dimeric RGD-containing peptidomimetics with longer linkers have been created to increase the flexibility between integrin binding motifs [45],[51]. In one study, a RGD dimer with a flexible glycine linker exhibited a 2-fold increase in affinity compared to a RGD dimer without a flexible linker (IC_50_ = 63±nM versus 102±5 nM, respectively) [52]. A ^64^Cu-labeled version of this dimer exhibited increased tumor uptake in U87MG tumor-bearing mice compared to a dimer without the linker (6.43±1.22%ID/g versus 3.83±0.22%ID/g, respectively) [52]. Higher-order multivalent compounds, such as RGD tetramers and octamers, have also exhibited higher binding and greater effects on tumor uptake compared to dimeric compounds [46], [53], although liver and kidney uptake were also increased.

^64^Cu-DOTA-knottin 3-4A showed moderate serum stability, with ∼30% of the intact probe remaining after 24 h. Intact probe was excreted in the urine, but showed substantial degradation in the tumor, liver, and kidneys at 1 h post injection. In contrast, ^64^Cu-DOTA-knottin 2.5D had much greater stability, with 82% intact probe remaining after incubation in serum for 24 h, and 90% and 88% intact probe recovered from the tumor and kidneys, respectively, at 1 h post injection [23]. The reduced stability of ^64^Cu-DOTA-knottin 3-4A compared to ^64^Cu-DOTA-knottin 2.5D is likely due to the replacement of a 5-amino acid loop (GPNGF) with a 10-amino acid loop (REARGDMPRT), potentially rendering the peptide more susceptible to cleavage with proteases. We detected the presence of intact probe by radio-HPLC as it is highly sensitive, and any unfolding or change in the chemical nature of the knottin peptide would be readily evident in the chromatograms. However functional read-outs of stability would also be useful in future studies to determine if degradation observed by reversed-phase HPLC correlates with functional activity. Despite its higher binding affinity to U87MG cells, the instability of ^64^Cu-DOTA-knottin 3-4A most likely influenced its tissue biodistribution, and is one explanation for why enhanced tumor uptake was not observed with this probe compared to ^64^Cu-DOTA-knottin 2.5D. In future studies, alternative knottin mutants isolated from our library screens could be analyzed for the effects of amino acid sequence on serum and metabolic stability, or D-amino acids could be explored in Loop 3 to improve its resistance to proteases. However, many factors play a role in tumor uptake and non-target tissue biodistribution of molecular imaging agents, including blood clearance rate, probe stability and metabolism, organ clearance route, tumor washout rate, receptor binding affinity, and receptor binding specificity. For example, we and others have shown a correlation between receptor binding affinity and tumor uptake using polypeptide-based probes [23], [54]. In addition, a computational model was recently used to understand and predict the interplay between molecular size, affinity, and tumor uptake, and correlated with experimental observations from the literature [55]. Using small peptidomimetics, correlations between higher-order multivalency, receptor binding affinity, and tumor uptake have also been uncovered [44], [45]. In contrast, recent studies have shown decreased tumor uptake with dimeric polypeptides compared to their monomeric counterparts, perhaps due to reduced tumor penetration and diffusion that occurs as protein size increases [41], [56], [57]. In the future, protein engineering will continue to generate additional tools to help illuminate the contribution of size, chemical composition, binding affinity, avidity, and stability to *in vivo* imaging performance, namely pharmacokinetics and tissue biodistribution.

In summary, we further demonstrate the versatility of EETI-II as a molecular scaffold for polypeptide engineering. To our knowledge, this is the first example where two functional loops of a knottin peptide have been engineered against an exogenous target. We observed remarkable tolerance to loop length and sequence diversity as the resulting 38 amino acid EETI-II mutant contained 21 non-native amino acid residues distributed across two loops. While serum and metabolic stability were reduced by these mutations, a radiolabeled version of this knottin peptide exhibited favorable properties for *in vivo* molecular imaging applications, namely high target binding affinity, relatively high T/B and T/M ratios, and low uptake and retention in non-target tissue. These results have important implications for future polypeptide engineering efforts, where multiple loops of a knottin peptide could be evolved to bind with high affinity against a molecular target of interest.

## Materials and Methods

### Materials, Cell Lines, and Reagents

U87MG glioblastoma cells and K562 leukemia cells were obtained from American Type Culture Collection (Manassas, VA), and integrin-transfected K562 cells [29] were provided by Scott Blystone (SUNY Upstate Medical University). Detergent-solubilized α_v_β_3_ integrin, octyl-beta-D-glucopyranoside formulation, was purchased from Millipore (Billerica, MA). ^125^I-labeled echistatin and c(RGDyK) were obtained from Amersham Biosciences (GE Healthcare, Piscataway, NJ), and Peptides International (Louisville, KY), respectively. Phosphate buffered saline (PBS) was purchased from Invitrogen (Carlsbad, CA). All other chemicals were obtained from Thermo Fisher Scientific (Pittsburgh, PA) unless otherwise specified. Selective SD-CAA media contained 20 g/L glucose, 6.7 g/L yeast nitrogen base without amino acids, 5.4 g/L Bacto casamino acids. SG-CAA media was identical except glucose was replaced by galactose. Integrin binding buffer (IBB) was composed of 25 mM Tris pH 7.4, 150 mM NaCl, 2 mM CaCl_2_, 1 mM MgCl_2_, 1 mM MnCl_2_, and 0.1% bovine serum albumin (BSA).

### Library Synthesis and Screening

The open reading frame encoding for EETI-II knottin 2.5D, with the RGD motif scrambled to RDG, was generated by overlap extension PCR. Loop 3 of this mutant (amino acids GPNGF) was substituted with the sequences XXXRGDXXX, XXXRGDXXXX, and XXXRGDXXXXX, where X =  any possible amino acid, by overlap extension PCR (Table S1). Positions for randomization were constructed using varying numbers of NNS degenerate codons, where N = A, T, C, or G and S = C or G, which codes for all possible 20 amino acids and the TAG stop codon. The assembly PCR products were amplified using primers with overlap to the pCT yeast display plasmid upstream or downstream of the NheI and BamHI restriction sites, respectively (Table S1). For each library, ∼40 µg of DNA insert and 4 µg of linearized pCT vector were electroporated into the *S. cerevisiae* strain EBY100 by homologous recombination as described [58]. The three libraries (∼5–7×10^6^ transformants each) were combined for a total potential diversity of ∼2×10^7^ clones as estimated by serial dilution plating and colony counting. Libraries were grown in SD-CAA media and induced for yeast cell surface display of knottin peptides in SG-CAA media as described [59].

For library screening, various concentrations of detergent-solubilized α_v_β_3_ integrin were added to yeast suspended in IBB for 2 h at room temperature. Next, a 1∶250 dilution of chicken anti-cMyc IgY antibody (Invitrogen) was added for 1 h at 4°C. The cells were washed with ice-cold IBB and incubated with a 1∶25 dilution of fluorescein-conjugated anti-α_v_ integrin antibody (mAb 13C2, Millipore) and a 1∶100 dilution of Alexa 555-conjugated goat anti-chicken IgG secondary antibody (Invitrogen) for 30 min at 4°C. Cells were washed as above and α_v_β_3_ integrin binders were isolated using a Becton Dickinson FACSVantage SE instrument (Stanford FACS facility). For the first round of sorting, approximately 1×10^8^ yeast clones were analyzed for binding to 100 nM α_v_β_3_ integrin. Collected yeast cells were cultured, induced for expression, and sorted by subsequent rounds of FACS to obtain an enriched population of integrin binders. To increase sort stringency, integrin concentrations were successively decreased to 2 nM in later sort rounds, and a diagonal sort gate was used to isolate yeast cells with enhanced integrin binding (FITC fluorescence) for a given protein expression level (Alexa 555 fluorescence). In between sorting rounds, cells were analyzed by dual-color flow cytometry using a BD FACSCalibur and CellQuest software (Becton Dickinson). Finally, an “off-rate” sort was performed by incubating yeast with 2 nM α_v_β_3_ integrin, followed by a 4 h unbinding step performed in the presence of 125 nM EETI-II knottin 2.5D competitor. A summary of the library sorting details is presented in Table S2. Plasmid DNA was recovered using a Zymoprep kit (Zymo Research Corporation, Orange, CA), amplified in XL-1 blue supercompetent *E. coli* cells (Stratagene/Agilent Technologies, Santa Clara, CA) and sequenced (MCLAB, South San Francisco, CA).

### Knottin Peptide Synthesis and Folding

Knottin peptides were synthesized on a CS Bio CS336 instrument using Fmoc-based solid phase peptide synthesis with Rink amide resin (CS Bio Company). Fmoc groups were removed with 20% piperidine in *N,N*-dimethylformamide (DMF). Amino acid coupling was performed using HOBt/diisopropylcarbodiimide chemistry in DMF. After synthesis, side-chain deprotection and resin cleavage was achieved by addition of a 94∶2.5∶2.5∶1 (v/v) mixture of trifluoroacetic acid (TFA)/triisopropylsilane/ethanedithiol/water for 2 h at room temperature. The crude product was precipitated with cold anhydrous diethyl ether, and purified by reversed-phase HPLC using a Varian Prostar instrument and Vydac C_18_ columns. Linear gradients of 90% acetonitrile in water containing 0.1% (v/v) TFA were used for all peptide purifications, which were monitored at an absorbance of 220 nm. Peptide purity was analyzed by analytical scale reversed-phase HPLC using a Vydac C_18_ column. Molecular masses were determined by matrix-assisted laser desorption/ionization time-of-flight mass spectrometry (MALDI-TOF MS) on a Perseptive Voyager-DE-RP Biospectrometry instrument (Stanford Protein and Nucleic Acid facility) (Figure S1 and Table S3). Folding reactions were performed by incubating peptides with 2.5 mM reduced glutathione and 20% dimethlysulfoxide (v/v) in 0.1 M ammonium bicarbonate, pH 9 with gentle rocking overnight. The final oxidized product was purified by reversed-phase HPLC as described above and lyophilized. Purified peptides were dissolved in water, and concentrations were determined by amino acid analysis (AAA Service Laboratory, Damascus, OR). Peptide purity and molecular masses were determined by analytical scale reversed-phase HPLC and MALDI-TOF MS (Figure S1 and Table S3). Purity was determined to be greater than 95%.

### Cell Surface Competition Binding Assay

Competition binding assays were performed as previously described [33], [60] to measure the relative binding affinities of engineered knottin peptides and echistatin. Briefly, 2×10^5^ U87MG or K562 cells were incubated with 0.06 nM ^125^I-labeled echistatin and varying concentrations of peptides in IBB at room temperature for 3 h. The cell-bound radioactivity remaining after washing was determined by gamma-counting. Half-maximal inhibitory concentration (IC_50_) values were determined by non-linear regression analysis using KaleidaGraph software (Synergy Software), and are presented as the average of experiments performed on three separate days.

### Cell Adhesion Assay

Cell adhesion assays were performed using Cytomax fibronectin- and vitronectin-coated strips (Millipore) as previously described [8], [61] and according to the manufacturer's protocol. Briefly, coated strips were rehydrated with PBS. Varying concentrations of peptides were added to 10^5^ U87MG cells in 100 µL of IBB, incubated for 2 h at 37°C, 5% CO_2_, and washed with Dulbecco's PBS (DPBS, Invitrogen). Remaining adherent cells were incubated with 100 µL of 0.2% crystal violet and 10% ethanol for 5 min at room temperature, washed in DPBS and solubilized with 100 µL/well of a 50∶50 mixture of 100 mM sodium phosphate, pH 4.5 and ethanol for 5 min. The absorbance at 600 nm was measured using a SPECTRAmax PLUS (Molecular Devices) microtiter plate reader. IC_50_ values were determined by non-linear regression analysis using KaleidaGraph software, and are the average of experiments performed on three separate days. Data was normalized against samples containing no competing peptide and background signal from uncoated wells.

### Chemical Conjugation of DOTA and ^64^Cu Radiolabeling

Knottin peptides were reacted with a 5-fold molar excess of *N*-hydroxysuccinimide ester-activated DOTA (DOTA-NHS ester; Macrocyclics, Dallas, TX) in DMF containing 2% *N,N*-diisopropylethylamine for 0.5 h to yield DOTA-knottin 3-4A. The DOTA-conjugated knottin peptide was purified by reversed-phase HPLC. Molecular masses were determined by MALDI-TOF MS and peptide products were analyzed by analytical scale reversed-phase HPLC (Figure S1 and Table S3). For each radiolabeling reaction, approximately 10–25 µg of DOTA-knottin 3-4A was incubated with 1–2 mCi ^64^CuCl_2_ (University of Wisconsin-Madison, Madison, WI) in 0.1 N sodium acetate (pH = 6) for 1 h at 42°C. The radiolabeled peptide was purified using a PD-10 column (Amersham Biosciences/GE Healthcare), eluted with PBS (pH 7.4), and passed through a 0.22 µm filter for animal experiments. The radiochemical purity, determined as the ratio of the main product peak to other peaks, was determined by HPLC to be >95%. The radiochemical yield, determined as the ratio of final activity of the product over the starting activity used for the reaction, was usually over 80%. The specific activity of the probe was ∼500 Ci/mmol.

### Tumor Model

Animal procedures were carried out according to a protocol approved by Stanford University Administrative Panels on Laboratory Animal Care (APLAC Protocol 11580). U87MG glioblastoma cells were maintained at 37°C/5% CO_2_ in Dulbecco's Modified Eagle Medium, 10% heat-inactivated fetal bovine serum, and penicillin-streptomycin (all from Invitrogen). Female athymic nude mice (nu/nu), obtained at 4–6 weeks of age (Charles River Laboratories, Inc., Wilmington, MA), were injected subcutaneously in the right or left shoulder with ∼10^7^ U87MG cells suspended in 100 µL of PBS. Mice were used for *in vivo* imaging studies when their tumors reached approximately 10 millimeters in diameter.

### MicroPET Imaging of Tumor Xenografts

U87MG tumor-bearing mice (n = 3 or more for each probe) were injected with ∼100 µCi (∼0.5 nmol) of probe via the tail vein and imaged with a microPET R4 rodent model scanner (Siemens Medical, Knoxville, TN) using 5 min static scans. Images were reconstructed by a two-dimensional ordered expectation maximum subset algorithm and calibrated as previously described [53]. ROIs were drawn over the tumor on decay-corrected whole body images using ASIPro VM software (Siemens Medical). ROIs were converted to counts/g/min, and %ID/g values were determined assuming a tissue density of 1 g/mL. No attenuation correction was performed. For the blocking experiments, mice were co-injected with 330 µg (∼0.5 µmol) of unlabeled c(RGDyK), a pentapeptide that binds to α_v_β_3_ and α_v_β_5_ integrins [8], [62] at a similar receptor binding site as the engineered knottin peptides.

### *In Vivo* Biodistribution Studies

Anesthetized nude mice bearing U87MG tumor xenografts were injected with ∼50–100 µCi (∼0.25–0.5 nmol) of ^64^Cu-DOTA-knottin 3-4A via the tail vein, and were euthanized at 1, 4, and 24 h post injection. Blood, bone, brain, heart, kidney, liver, lung, muscle, pancreas, skin, spleen, and tumor tissue were removed and weighed, and their radioactivity levels (counts per minute) were measured by gamma counting. The radioactivity uptake in the tumor and normal tissue was reported as the percent injected dose per gram (%ID/g) of tissue and represent the mean and standard deviation of experiments performed on three mice. For each mouse, the activity of tissue samples was calibrated against a pre-measured aliquot of the radiotracer and normalized to the whole bodyweight and to the residual radioactivity present in the tail. To test integrin-targeting specificity, U87MG tumor-bearing mice (n = 3) were co-injected with ^64^Cu-DOTA-knottin 3-4A and 330 µg (∼0.5 µmol) of unlabeled c(RGDyK), and biodistribution of the radiolabeled peptide was determined 1 h after injection.

### *In Vitro* and *In Vivo* Stability

Aliquots containing up to 400 µCi of ^64^Cu-DOTA-knottin 3-4A were incubated in 50% mouse serum for up to 24 h. At various time points, samples were mixed with an equal volume of 99.9% water/0.1% TFA, further acidified with ∼1% (v/v) TFA, and centrifuged to remove precipitants. Soluble fractions were filtered with a 0.2 µm microcentrifuge filter (Corning Costar Spin-X filter) and analyzed by reversed-phase radio-HPLC using a Vydac C_18_ column. For metabolic stability analysis, anesthetized nude mice bearing U87MG tumor xenografts were injected with 200–400 µCi of ^64^Cu-DOTA-knottin 3-4A via the tail vein, and were euthanized at 1 h post injection. Tumor, kidney, and liver tissues were homogenized with a mortar/pestle in 1 ml DMF. The lysates were acidified, centrifuged, and filtered using a 0.2 µm Costar Spin-X microcentrifuge filter to isolate soluble metabolites. Filtrates were injected onto a Vydac C_18_ column and analyzed by reversed-phase HPLC. Fractions were collected every 1 min and analyzed by gamma counting to determine the counts per minute (cpm). Urine samples were collected 1 h post injection and were also analyzed by radio-HPLC. The intact radiotracer elutes at approximately 15 min, and the major hydrophilic metabolites and free copper elute with the column dead volume.

### Statistical Analysis

All data are presented as the average value ± the SD of at least 3 independent measurements. Statistical analysis for animal studies and binding studies were performed by two factor ANOVA without replication analysis using Microsoft Excel. Significance was assigned for *p* values of <0.05.

## Supporting Information

Table S1 Primers for library construction. Primers for PCR assembly of EETI-II libraries, and primers for amplification of assembly products and homologous recombination in yeast.
(DOCX)

Table S2 Library screening details. Nine sort rounds were performed against varying concentrations of α_v_β_3_ integrin. In round 9, an “off-rate” sort was performed by incubating yeast with 2 nM α_v_β_3_ integrin, followed by a 4 h unbinding step in the presence of 125 nM EETI-II knottin 2.5D competitor.
(DOCX)

Table S3 Mass spectrometry. MALDI-TOF-MS of the reduced, oxidized (folded), or DOTA conjugated form of knottin peptide 3-4A. The [M+H]^+^ state is indicated.
(DOCX)

Figure S1 Reversed-phase HPLC chromatograms of knottin synthesis, folding, and DOTA conjugation. Representative analytical scale HPLC traces of knottin 3-4A, indicated by asterisks. (**A**) Crude material from peptide synthesis, (**B**) Folding reaction, (**C**) Purified oxidized (folded) peptide, and (**D**) Purified DOTA conjugated peptide. (**A**) Gradient  = 20–50% solvent B (90% acetonitrile/10% water/0.1% trifluoroacetic acid) over 30 min, and (**B–D**) Gradient  = 10–50% solvent B over 30 min.
(TIF)

Figure S2 Competition binding to integrin receptors expressed on K562 cell lines. Varying concentrations of unlabeled peptides were incubated with ^125^I-echistatin and allowed to compete for binding to cell surface receptors present on (**A**) untransfected K562 cells, which express α_5_β_1_ integrin, or K562 cells stably transfected with (**B**) α_v_β_3_ integrin, (**C**) α_v_β_5_ integrin, or (**D**) α_iib_β_3_ integrin. Percent of ^125^I-echistatin bound to the cell surface is plotted versus the concentration of unlabeled echistatin (•), knottin 3-4A (⧫), or knottin 3-4C (▪). Data shown are the average of three replicates performed on different days and error bars represent standard deviations. IC_50_ values are summarized in Table 1.
(TIF)

Figure S3 Cell binding and uptake assay. To measure cell binding and uptake, 5×10^5^ U87MG cells were suspended in 50 µL IBB and incubated with ^64^Cu-DOTA-knottin 3-4A (1.5 µCi/tube in 100 µL IBB) at 37°C for 30, 60, and 120 min. Cells were washed three times with ice-cold PBS and pelleted by centrifugation. The radioactivity of the cell pellet was measured with a gamma counter (PerkinElmer 1470, Waltham, MA). Cell binding and uptake of ^64^Cu-DOTA-knottin 3-4A was expressed as the percentage of added radioactivity. Target specificity was further determined by blocking experiments where 1 µg unlabeled c(RGDyK) pentapeptide was co-incubated with ^64^Cu-DOTA-knottin 3-4A (block). Experiments were performed in triplicate.
(TIF)
