# Supplementary material for: Functional Mutation of Multiple Solvent-Exposed Loops in the Ecballium elaterium Trypsin Inhibitor-II Cystine Knot Miniprotein
Source: PLoS One. 2011 Feb 18;6(2):e16112. doi: 10.1371/journal.pone.0016112 (PMC3041754; doi:10.1371/journal.pone.0016112)
Supplement: Table S1 — Primers for library construction. Primers for PCR assembly of EETI-II libraries, and primers for amplification of assembly products and homologous recombination in yeast. (DOCX) [file pone.0016112.s001.docx]

**Table S1. Primers for library construction.**

| Primers for PCR assembly of EETI-II libraries. Restriction sites are underlined. |
| --- |
| Forward: |
| 5’-GCTAGCGGTTGTCCACAAGGTAGAGATGGTTGGGCTCCAACTTCTTGTTCTCAAGATTCTGATTGTTTGGCTGGTTGT-3’ |
| Reverse: |
| XXXRGDXXX  5’-GGATCCAGAACCACCACCACASNNSNNSNNATCACCTCTSNNSNNSNNACAAACACAACCAGCCAAACAATCAGA-3’ |
|  |
| XXXRGDXXXX  5’-  GGATCCAGAACCACCACCACASNNSNNSNNSNNATCACCTCTSNNSNNSNNACAAACACAACCAGCCAAACAATCAGA-3’ |
| XXXRGDXXXXX  5’-  GGATCCAGAACCACCACCACASNNSNNSNNSNNSNNATCACCTCTSNNSNNSNNACAAACACAACCAGCCAAACAATCAGA-3’ |
|  |
| Primers for amplification of assembly products and homologous recombination in yeast. Restriction sites are underlined. Note that an extra Gly-Gly-Ser was added before the C-terminal c-myc tag to help reduce steric hindrance. |
| Forward: |
| 5’-TGGTGGTTCTGGTGGTGGTGGTTCTGGTGGTGGTGGTTCTGCTAGCGGTTGTCCACAAGG-3’ |
| Reverse: |
| 5’-CGAGCTATTACAAGTCCTCTTCAGAAATAAGCTTTTGTTCGGATCCAGAACCACCACCACA–3’ |
