# Supplementary material for: Functional Mutation of Multiple Solvent-Exposed Loops in the Ecballium elaterium Trypsin Inhibitor-II Cystine Knot Miniprotein
Source: PLoS One. 2011 Feb 18;6(2):e16112. doi: 10.1371/journal.pone.0016112 (PMC3041754; doi:10.1371/journal.pone.0016112)
Supplement: Table S2 — Library screening details. Nine sort rounds were performed against varying concentrations of αvβ3 integrin. In round 9, an “off-rate” sort was performed by incubating yeast with 2 nM αvβ3 integrin, followed by a 4 h unbinding step in the presence of 125 nM EETI-II knottin 2.5D competitor. (DOCX) [file pone.0016112.s002.docx]

**Table S2. Library screening details.** Nine sort rounds were performed against varying concentrations of α_v_β_3_ integrin. In round 9, an “off-rate” sort was performed by incubating yeast with 2 nM α_v_β_3_ integrin, followed by a 4 h unbinding step in the presence of 125 nM EETI-II knottin 2.5D competitor.

| **Sort Round** | **Sort Conditions** | **# Cells Sorted** | **# Cells Collected** | **% Collected** |
| --- | --- | --- | --- | --- |
| 1 | 100 nM integrin | 1.5 x 10^8^ | 2.2 x 10^6^ | 1.5% |
| 2 | 50 nM integrin | 1.8 x 10^7^ | 2.5 x 10^5^ | 1.4% |
| 3 | 50 nM integrin | 1.6 x 10^6^ | 5.9 x 10^4^ | 3.7% |
| 4 | 25 nM integrin | 1.6 x 10^6^ | 4.6 x 10^4^ | 2.9% |
| 5 | 25 nM integrin | 1.1 x 10^6^ | 2.3 x 10^4^ | 2.1% |
| 6 | 10 nM integrin | 7.4 x 10^5^ | 6.1 x 10^3^ | 0.8% |
| 7 | 10 nM integrin | 1.0 x 10^6^ | 5.5 x 10^3^ | 0.6% |
| 8 | 2 nM integrin | 1.0 x 10^6^ | 5.7 x 10^3^ | 0.6% |
| 9 | 2 nM integrin,  4 h “off” | 1.0 x 10^6^ | 7.0 x 10^3^ | 0.7% |
