# Supplementary material for: Functional Mutation of Multiple Solvent-Exposed Loops in the Ecballium elaterium Trypsin Inhibitor-II Cystine Knot Miniprotein
Source: PLoS One. 2011 Feb 18;6(2):e16112. doi: 10.1371/journal.pone.0016112 (PMC3041754; doi:10.1371/journal.pone.0016112)
Supplement: Table S3 — Mass spectrometry. MALDI-TOF-MS of the reduced, oxidized (folded), or DOTA conjugated form of knottin peptide 3-4A. The [M+H]+ state is indicated. (DOCX) [file pone.0016112.s003.docx]

**Table S3. Mass spectrometry.** MALDI-TOF-MS of the reduced, oxidized (folded), or DOTA conjugated form of knottin peptide 3-4A. The [M+H]^+^ state is indicated.

| **Peptide** | **Expected Mass (Da)** | **Observed Mass (Da)** |
| --- | --- | --- |
| Reduced | 3944.6 | 3945.4 |
| Oxidized | 3938.6 | 3939.1 |
| DOTA-knottin 3-4A | 4326.0 | 4326.0 |
