# Supplementary figures and images for: Functional Mutation of Multiple Solvent-Exposed Loops in the Ecballium elaterium Trypsin Inhibitor-II Cystine Knot Miniprotein
Source: PLoS One. 2011 Feb 18;6(2):e16112. doi: 10.1371/journal.pone.0016112 (PMC3041754; doi:10.1371/journal.pone.0016112)

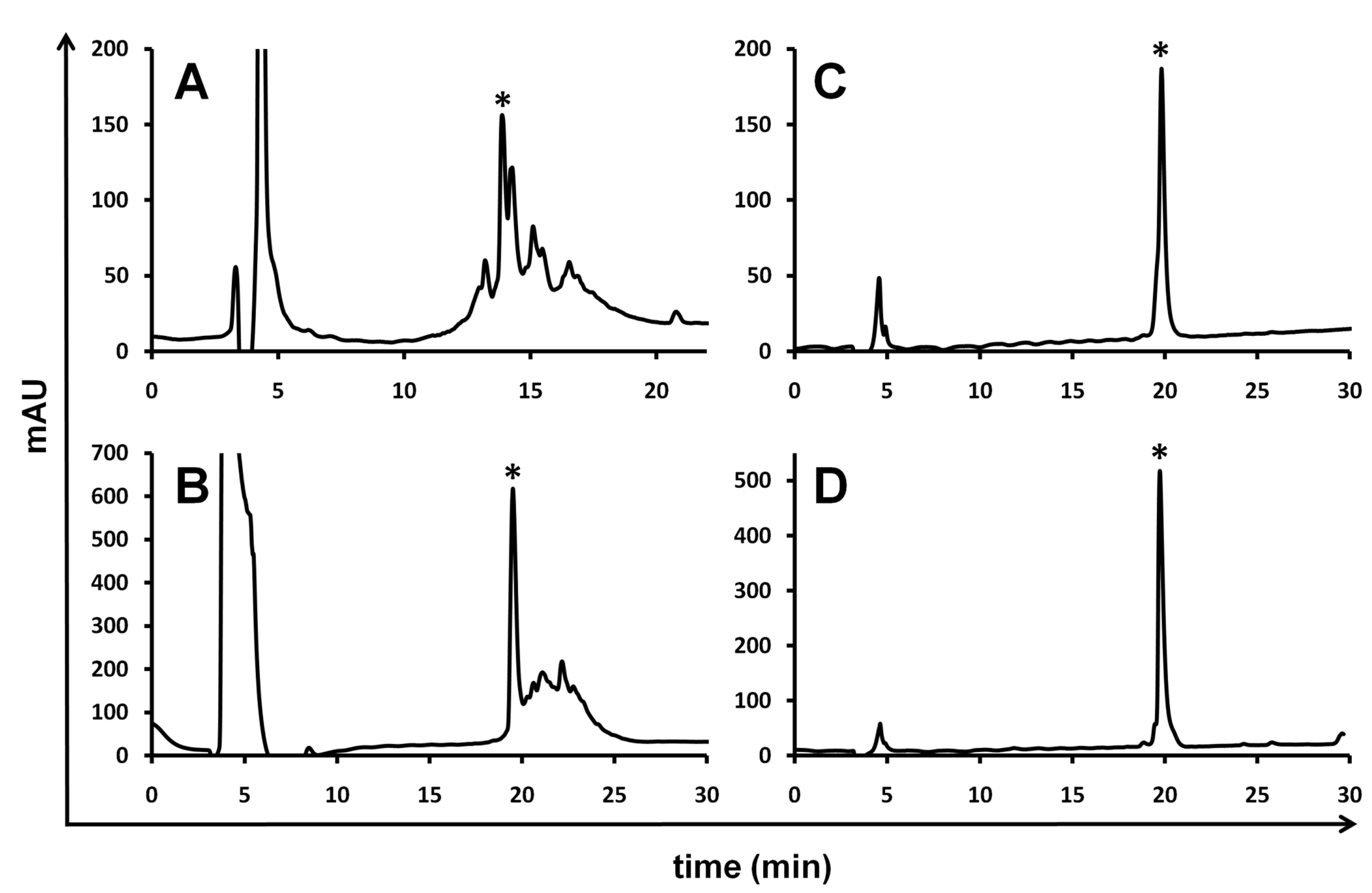

Supplement: Figure S1 — Reversed-phase HPLC chromatograms of knottin synthesis, folding, and DOTA conjugation. Representative analytical scale HPLC traces of knottin 3-4A, indicated by asterisks. (A) Crude material from peptide synthesis, (B) Folding reaction, (C) Purified oxidized (folded) peptide, and (D) Purified DOTA conjugated peptide. (A) Gradient = 20–50% solvent B (90% acetonitrile/10% water/0.1% trifluoroacetic acid) over 30 min, and (B–D) Gradient = 10–50% solvent B over 30 min. (TIF) [file pone.0016112.s004.tif]

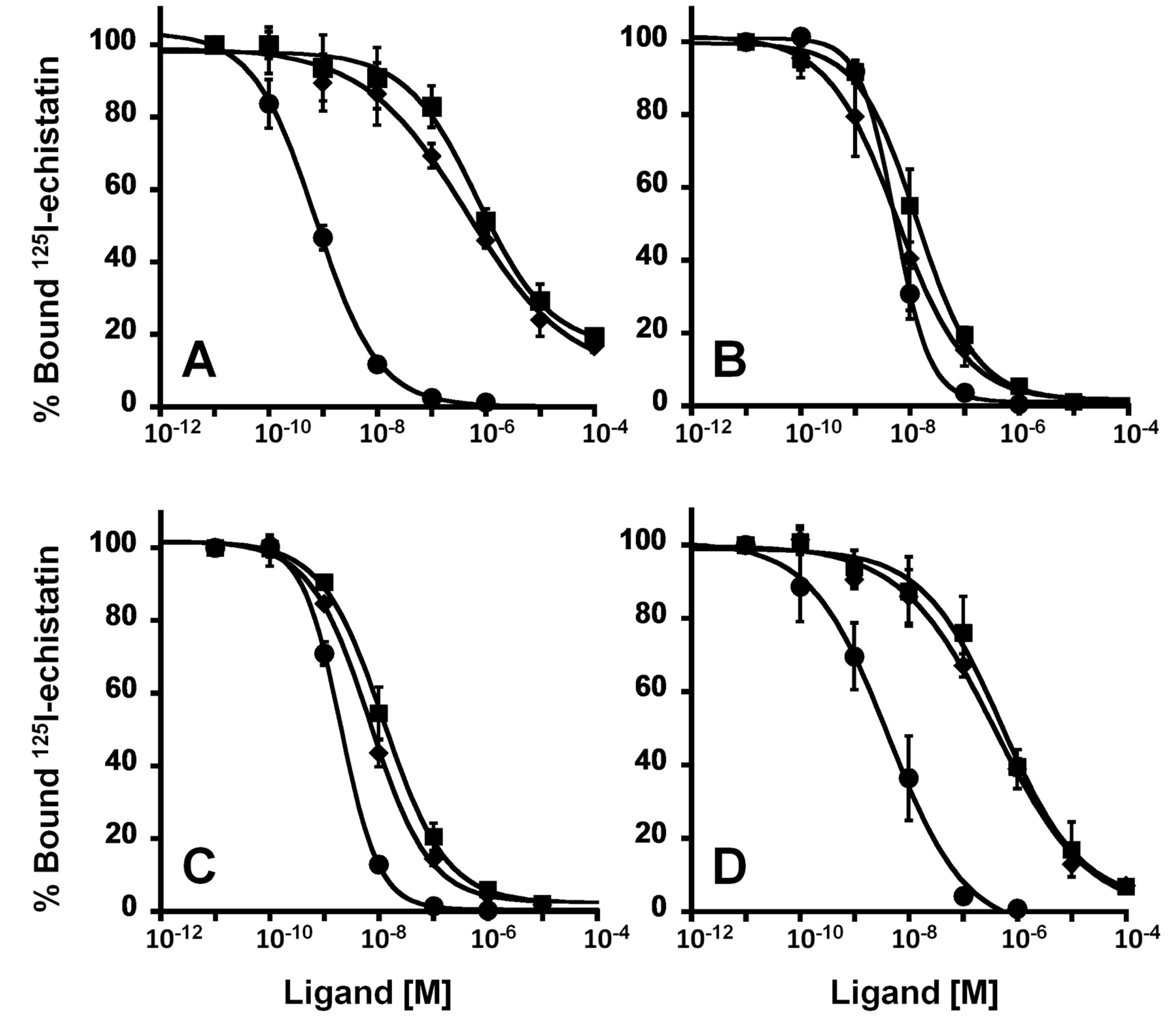

Supplement: Figure S2 — Competition binding to integrin receptors expressed on K562 cell lines. Varying concentrations of unlabeled peptides were incubated with 125I-echistatin and allowed to compete for binding to cell surface receptors present on (A) untransfected K562 cells, which express α5β1 integrin, or K562 cells stably transfected with (B) αvβ3 integrin, (C) αvβ5 integrin, or (D) αiibβ3 integrin. Percent of 125I-echistatin bound to the cell surface is plotted versus the concentration of unlabeled echistatin (•), knottin 3-4A (⧫), or knottin 3-4C (▪). Data shown are the average of three replicates performed on different days and error bars represent standard deviations. IC50 values are summarized in Table 1. (TIF) [file pone.0016112.s005.tif]

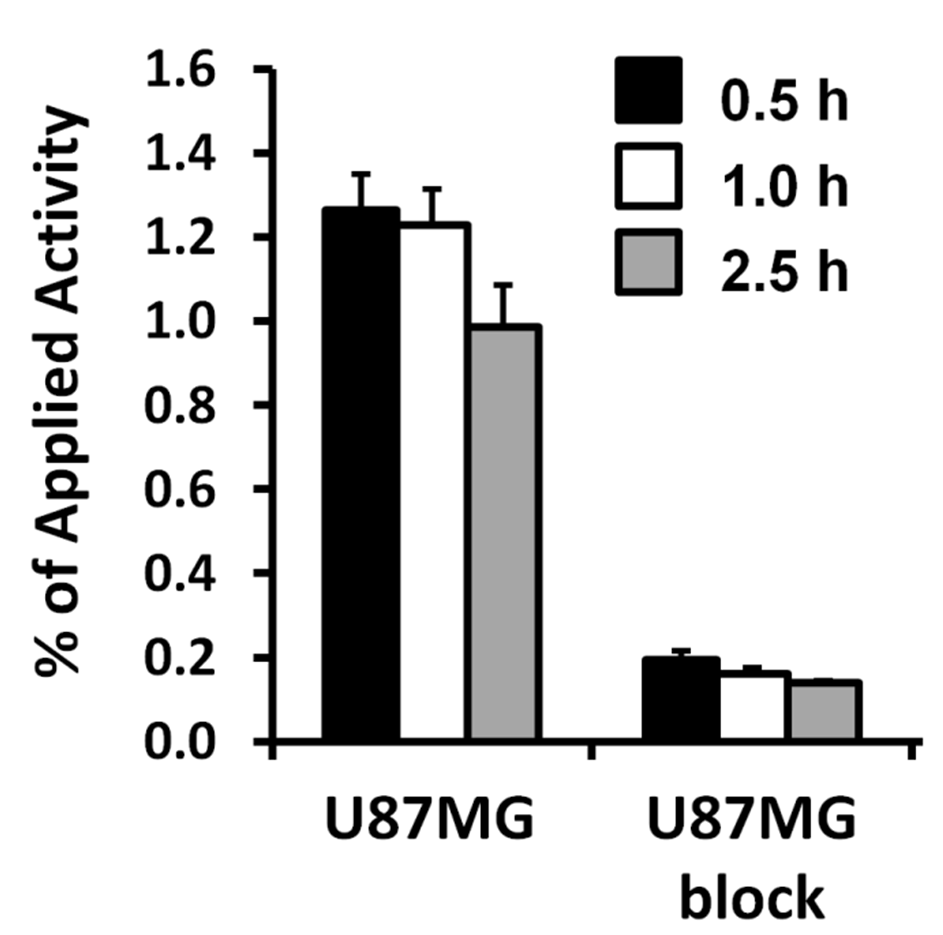

Supplement: Figure S3 — Cell binding and uptake assay. To measure cell binding and uptake, 5×10^5^ U87MG cells were suspended in 50 µL IBB and incubated with 64Cu-DOTA-knottin 3-4A (1.5 µCi/tube in 100 µL IBB) at 37°C for 30, 60, and 120 min. Cells were washed three times with ice-cold PBS and pelleted by centrifugation. The radioactivity of the cell pellet was measured with a gamma counter (PerkinElmer 1470, Waltham, MA). Cell binding and uptake of 64Cu-DOTA-knottin 3-4A was expressed as the percentage of added radioactivity. Target specificity was further determined by blocking experiments where 1 µg unlabeled c(RGDyK) pentapeptide was co-incubated with 64Cu-DOTA-knottin 3-4A (block). Experiments were performed in triplicate. (TIF) [file pone.0016112.s006.tif]
